# MTA-Enriched Polymeric Scaffolds Enhanced the Expression of Angiogenic Markers in Human Dental Pulp Stem Cells

**DOI:** 10.1155/2022/7583489

**Published:** 2022-02-21

**Authors:** Mahdieh Alipour, Zahra Aghazadeh, Mehdi Hassanpour, Marjan Ghorbani, Roya Salehi, Marziyeh Aghazadeh

**Affiliations:** ^1^Dental and Periodontal Research Center, Faculty of Dentistry, Tabriz University of Medical Sciences, Tabriz, Iran; ^2^Stem Cell Research Center, Tabriz University of Medical Sciences, Tabriz, Iran; ^3^Department of Oral Medicine, Faculty of Dentistry, Tabriz University of Medical Sciences, Tabriz, Iran; ^4^Nutrition Research Center, Tabriz University of Medical Sciences, Tabriz, Iran; ^5^Department of Medical Nanotechnology, Faculty of Advanced Medical Sciences, Tabriz University of Medical Sciences, Tabriz, Iran

## Abstract

Revascularization of the pulp tissue is one of the fundamental processes and challenges in regenerative endodontic procedures (REPs). In this regard, the current study is aimed at synthesizing the mineral trioxide aggregate- (MTA-) based scaffolds as a biomaterial for REPs. Poly (*ε*-caprolactone) (PCL)/chitosan (CS)/MTA scaffolds were constructed and evaluated by FTIR, SEM, XRD, and TGA analyses. Proliferation and adhesion of human dental pulp stem cells (hDPSCs) were assessed on these scaffolds by scanning electron microscopy (SEM) and MTT assays, respectively. The expression of angiogenic markers was investigated in gene and protein levels by real-time PCR and western blotting tests. Our results indicated that the obtained appropriate physicochemical characteristics of scaffolds could be suitable for REPs. The adhesion and proliferation level of hDPSCs were significantly increased after seeding on PCL/CS/MTA scaffolds. The expression levels of VEGFR-2, Tie2, and Angiopoietin-1 genes were statistically increased on the PCL/CS/MTA scaffold. In support of these findings, western blotting results showed the upregulation of these markers at protein levels in PCL/CS/MTA scaffold (*P* < 0.05). The current study results suggested that PCL/CS/MTA scaffolds provide appropriate structures for the adhesion and proliferation of hDPSCs besides induction of the angiogenesis process in these cells.

## 1. Introduction

The treatment of necrotic and inflamed immature permanent teeth has been considered a challenging clinical situation [1]. Recently, root end barriers with mineral trioxide aggregate- (MTA-) based materials have been used in these cases. However, the limitations of this method are the difficulty of applying apical stops, preventing root development, and increasing the risk of tooth fracture [2, 3].

To overcome these limitations, regenerative endodontic procedures (REPs) have been introduced as a promising approach for the regeneration of the dentin-pulp complex [4–6]. Like tissue engineering (TE) fundamentals, scaffolds (as a carrier and conductive agent), stem cells (as renewal elements), and biofactors (as stimulators) are the main components in REP strategy. Moreover, in REPs, appropriate angiogenesis should be provided to regenerate pulp tissue, the continuation of root development, apical closure, and thickening of the dentinal walls [5, 7, 8]. Pulp tissue is a highly vascularized tissue surrounded by dentin. Due to this architecture of the dentin-pulp complex, the angiogenesis procedure during tissue regeneration is challenging [9].

Dental pulp stem cells (DPSCs) are multipotent stem cells in the cell-rich zone of the dentin-pulp complex, which are able to differentiate into various cell types and promote the regeneration process of pulp tissue [10, 11].

A suitable scaffold as another crucial part of REPs should construct a 3D microenvironment for cell infiltration, migration, adhesion, proliferation, and differentiation. Poly (*ε*-caprolactone) (PCL) is a synthetic biocompatible and biodegradable material with mineralization capacity, which is successfully used for dentin and bone tissue engineering [12, 13]. Besides PCL, chitosan (CS), generally derived from the shells of shrimp and other sea crustaceans, is a natural polysaccharide that is widely used as a scaffold in tissue engineering [14]. This cationic polymer has high hydrophilicity, biocompatibility, and biodegradability [15]. The ability of CS to induce mineralization, chelating, antimicrobial effects, and anti-inflammatory properties turn it into an efficient gradient in pulp tissue regeneration [16, 17]. Moreover, CS polymer combined with calcium phosphate particles leads to the formation of mineralized tissues [18].

MTA has been used in endodontic treatment since 1993. The applications of this calcium silicate-based material in endodontic include perforation treatment, apexification of immature teeth, and repair of root resorbing [19, 20]. This biomaterial can induce dentinogenesis in hDPSCs [21]. However, this biomaterial has a long setting time and difficult handling and manipulating procedure which are considered drawbacks [3]. Moreover, it does not have a porous and interconnected structure, which is essential for tissue engineering [22–24]. MTA is generally considered a barrier material, not a scaffold in endodontic treatment. MTA is not absorbable, and it is a cement-like material. Therefore, it seems that designing and fabrication of scaffold containing this biomaterial can provide a better structure for regenerative purposes in endodontics. Thus, the current study was conducted to construct PCL/CS/MTA scaffolds and evaluate their ability to induce angiogenesis in hDPSCs.

## 2. Materials and Methods

### 2.1. Materials

Poly (*ε*-caprolactone) (PCL), collagenase, and glutaraldehyde (25%) were purchased from Sigma-Aldrich Co. (Steinem, Germany). CS was supplied from Acros Chemical Co. Dimethyl sulfoxide (DMSO), chloroform, absolute methanol (MeOH), and acetic acid were obtained from Merck Chemical Co. Phosphate-buffered saline (PBS), fetal bovine serum (FBS), trypsin-EDTA solution, high-glucose Dulbecco's modified Eagle's medium (DMEM/HG), and penicillin/streptomycin were obtained from Gibco. 3-(4,5-Dimethylthiazol-2-yl-2,5-diphenyltetrazolium bromide) (MTT) powder was obtained from Invitrogen (Carlsbad, CA, USA). MTA (ProRoot MTA) was obtained from Dentsply (Tulsa, OK).

### 2.2. Preparation of Scaffolds

First, to prepare the 5 wt% PCL solution, PCL (70–90 kD, Sigma Aldrich) was dissolved in glacial acetic acid, and then, 2 wt% CS powder (250 kD, 85% deacetylated) was added to this solution. The blends were homogenized at 20,000 rpm in the presence of polyvinyl alcohol (PVA) as a surfactant for hours until a clear, viscous solution was obtained. To prepare the PCL/CS/MTA sample, MTA (30 wt %, versus total polymer weight) was added and homogenized using a homogenizer. Afterward, the solution was transferred into a mold and frozen overnight at -70°C. Then, the frozen scaffolds were lyophilized in a freeze-dryer for at least 2 days to completely remove the solvents.

### 2.3. Scaffold Evaluation and Characterizations

The intermolecular interaction between the MTA and PCL/CS and the phase composition of the scaffolds were evaluated by Fourier transform infrared spectroscopy (FTIR) and X-ray diffraction (XRD), respectively. The FTIR test was taken by Tensor 27 (Bruker, Germany), in the Faculty of Chemistry, Tabriz University. To prepare the samples, these specimens were firstly poured into a uniform powder with a ratio of one to one hundred pure KBr. Then, the powder was made to tablet, placed in special tubes, and spectra with a wavenumber of 400 to 4000 cm^−1^ were recorded. XRD test was conducted by Bruker Discover 8 X-ray diffractometer (Germany) at 40 kV and 40 mA using the Cu-K*α* radiation in a 2*θ* range from 5° to 78°. Also, thermogravimetric analysis (TGA) was performed by heating the fabricated scaffolds with a rate of 10°C/min from 30°C to 600°C under a nitrogen gas flow (TGA/SDTA851/Mettler Toledo, Spain).

Moreover, the *in vitro* degradation and swelling rate of fabricated scaffolds were evaluated. For degradation analysis, PCL/CS and PCL/CS/MTA scaffolds were immersed in PBS (pH 7.4) at 37°C for 72 h. The specimens were weighed at 0, 6, 12, 24, 48, and 72 h. The weight loss percentage was measured according to the following formula, where *W*_f_ and *W*_i_ referred to the weight of the scaffold sample and the initial weight at various hours, respectively.

(1) $$\begin{array}{c} \text{Weight loss }\left(\%\right)=\frac{W_{\text{f}}}{W_{\text{i}}}\times 100. \end{array}$$

The remaining weight was reported according to the following formula:

(2) $$\begin{array}{c} 100-\text{weight loss}\left(\%\right)=\text{weight remaining}. \end{array}$$

For analysis of swelling rate, PCL/CS and PCL/CS/MTA scaffolds were cut into 2*×*2cm^2^ pieces and immersed in deionized water (15 mL) and incubated at 37°C for 24 hours. At certain time intervals, the samples were removed from the solution, and then, to eliminate the absorbed water on the surface of samples, they were slightly pressed by the filter paper. The scaffolds were weighed before and after immersing to measure dried (*W*_1_) and wet (*W*_2_) weights. The swelling of synthesized scaffolds was calculated according to the following equation:

(3) $$\begin{array}{c} \text{Swelling }\left(\%\right)=\frac{W_{2}-W_{1}}{W_{\text{i}}}\times 100. \end{array}$$

### 2.4. Sterilization of Scaffolds

The PCL/CS and PCL/CS/MTA scaffolds were placed under UV light for 30 min and then immersed in a 70% ethanol solution for 20 min. After that, the ethanol was evaporated, and the samples were washed with PBS solution to remove the excess ethanol. The sterilized scaffolds were incubated in DMEM at 37°C for 24 h, as described previously [25].

### 2.5. Cell Isolation, Characterization, and Culture of hDPSCs

The isolation and characterization of hDPSCs were processed according to the described protocol in our previous study [25, 26]. Briefly, after obtaining informed consent from the patients or their parents, intact permanent teeth were extracted based on the orthodontic treatment plan. Extracted teeth were delivered to the laboratory, and after cleaning, a groove was cut around the teeth. Then, the dental pulps were removed by splitting the teeth along the groove. The pulp tissues were cut to small pieces by surgical scissors and subsequently were enzymatically digested via collagenase type I and dispase. After centrifuging, the cell suspensions were cultured in a 25 cc culture flask containing D-modified Eagle's medium/high glucose (DMEM/HG), 10% fetal bovine serum (FBS), 100 units/mL penicillin, and 100 mg/mL streptomycin. DPSCs were incubated at 37°C in an 85-95% humid atmosphere containing 5% CO_2_. The culture medium was refreshed every 72 h. According to the flow cytometry analysis in our previous study [25], more than 80% of the expanded cells expressed surface antigens of mesenchymal stem cells such as CD90, CD166, CD105, and CD73, while only less than 4% of them expressed differentiated cell epitopes including CD64, CD106, CD11b, and CD31. For this purpose, at almost 100% confluency in the third passage, DPSCs were divided into 6 fluorescence-activated cell sorting round-bottom tubes (Becton Dickinson Falcon, Sunnyvale, CA) at the density of 2 × 10^5^ cells per tube and then stained with immunoglobulin G–fluorescein isothiocyanate-conjugated or phycoerythrin-conjugated anti-CD105, CD90, CD166, CD73, CD64, CD106, CD11b, and CD31 (Beckman Coulter, Villepinte, France, 20 mL each). The tubes were incubated for 20 min in a dark place at room temperature. The supernatant was removed after washing and centrifuging, and cells were fixed with 1% formaldehyde. All data were evaluated using a Coulter Epics XL (Beckman Coulter) and assessed using EXPO32 ADC software (Beckman Coulter) and WinMDI version 2.8.

### 2.6. Scanning Electron Microscopy (SEM) Imaging

HDPSCs (third passage) were cultured in DMEM/HG amplified with 10% FBS and 1% penicillin/streptomycin. The exhausted culture medium was replaced every 3 days until cells reached 80% confluence, cultured on sterilized scaffolds.

The surface morphology of PCL/CS/MTA scaffolds and hDPSCs cultured on scaffolds was evaluated by SEM 3 days after seeding. Before assessing, hDPSCs were fixed in 2.5% glutaraldehyde as described in our previous study [27]. After fixation, scaffolds with and without cells were cut and coated by the nanometer-thick gold layer. FE-SEM 1430 vp (MIRA3 FEG-SEM, Tescan, Czech) was applied to sample imaging.

### 2.7. Cell Proliferation Analysis by the MTT Test

To evaluate the toxicity of scaffolds on hDPSCs, an MTT assay was performed; briefly, cells (third passage) were seeded on PCL/CS and PCL/CS/MTA scaffolds. The scaffolds were placed in 48-well plates and then seeded by 5000 cells per well. In order to culture cells on the scaffolds with the same size of each well, firstly, 100 *μ*L suspension of culture medium containing cells was added to the scaffolds. After incubating the plates for one hour, 300 *μ*L culture medium was added to each well. Cells cultured in a medium without scaffolds were considered the control group. The proliferation of hDPSCs in scaffolds was evaluated 3, 7, and 14 days after seeding. 100 *μ*L of MTT solution (5 mg/mL) was added to each well. The plates were incubated at 37°C and 5% CO_2_ for 4 hours. After that, the MTT/medium solution was replaced by 100 *μ*L DMSO. Then, 100 *μ*L of solution from each well was transferred to 96-well plates, and the absorbance of dissolving blue formazan crystals was determined at 570 nm by an ELISA reader.

### 2.8. Gene Expression by the Quantitative Real-Time PCR

The angiogenic modifications of cultured cells were evaluated 7 days after seeding of 1 × 10^6^ of hDPSCs on fabricated scaffolds. Scaffolds were cut in the shape of the cylinder with 14 mm in diameter and 2 mm in height and placed in the 6-well plates. Cells (third passage) were cultured on 6-well plates with the same amounts (without scaffolds) and considered the control group. Briefly, after cell culture with/without scaffolds in angiogenic medium, total RNA was extracted according to the manufacturer instruction by Ambion TRIzol buffer (Cat No. 15596-026, Invitrogen, USA), and the quality of obtained RNA was determined with NanoDrop (Thermo Scientific, Waltham, MA, USA). Next, the cDNA synthesis was performed using 1 *μ*g of the extracted RNA by cDNA synthesis kit (Yekta Tajhiz Azma, Tehran, Iran). Finally, the quantitative real-time PCR assay (qRT-PCR) was done by mixing SYBR green master mix (Yekta Tajhiz Azma, Tehran, Iran), synthesized cDNA, designed primers, and distilled water based on the manufacturer protocol. Designed primer sequences and melting temperature are offered in Table 1. Each data was repeated in three separate experiments and three times. Data analysis was performed by the Pfaffl method.

### 2.9. Protein Expression by Western Blot Analysis

The immunoblotting assay was used to measure the expression of angiogenic proteins, including VEGFR-2, Angiopoietin-1, and Tie2 proteins in seeded hDPSCs in PCL/CS/MTA scaffolds. Briefly, cells were lysed in ice-cold cell lysis buffer solution (NaCl, NP-40, and Tris–HCl) containing cocktail enzyme inhibitors. After that, cell lysates were prepared and subjected to blot analysis. Blots were incubated with each primary antibody (Angiopoietin-1: Cat No. sc-517593, *β*-Actin: Cat No. sc-47778, Tie2: Cat No. sc-293414, Santa Cruz Biotechnology, Santa Cruz, CA; VEGFR-2: Cat No. ab39256, Abcam) overnight at 4°C and secondary HRP-conjugated with anti-IgG antibody (Cat No. sc-2357, Santa Cruz Biotechnology, Inc.) for 1 h at room temperature. Immunopositive bands were visualized by an enhanced chemifluorescent labeling kit (Amersham Pharmacia Biotech, Piscataway, NJ).

### 2.10. Statistical Analysis

In this study, data were presented as mean ± SD and analyzed by one-way ANOVA and Tukey post hoc test using GraphPad Prism software (version 8.0, GraphPad, San Diego, CA, USA). *P* value < 0.05 was considered statistically significant. All experiments were carried out with the same dental pulp stem cell line in triplicates.

## 3. Results

### 3.1. Physicochemical and Morphologic Properties of PCL/CS/MTA Scaffolds

**Figure 1:**
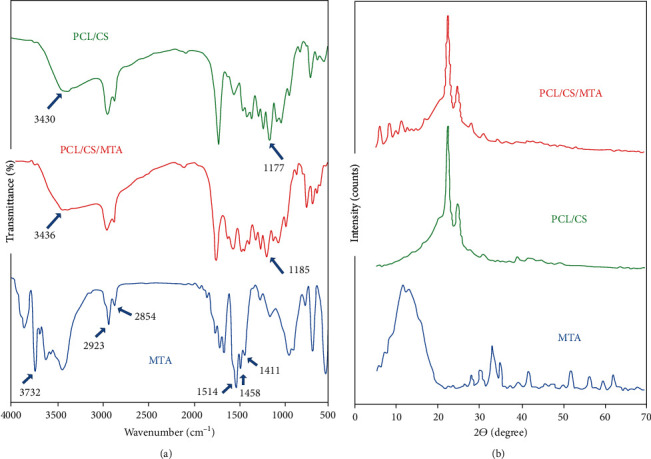
(a) FTIR spectra of PCL/CS, PCL/CS/MTA, and MTA. (b) XRD patterns for PCL/CS, PCL/CS/MTA, and MTA.

FTIR spectra of PCL/CS, PCL/CS/MTA, and MTA are demonstrated in Figure 1(a). The FTIR spectrum of MTA presented the specific bands of PO_4_^3-^ from stretching and bending mode (673 cm^−1^, 918 cm^−1^) and OH^−^ (673 cm^−1^, 3559 cm^−1^) from MTA [28, 29] and also CO_3_^2-^ (1458 cm^−1^) for carbonated MTA [29]. Moreover, the bands for SiO_4_^4-^ (526 cm^−1^, 975 cm^−1^) and OH^−^ (3857 cm^−1^, 3732 cm^−1^, 2923 cm^−1^, and 2854 cm^−1^) specific for calcium silicates hydrates were also present on the FTIR spectrum [30, 31]. The specific bands of CO_3_^2-^ from CaCO_3_ (1411 cm^−1^, 1514 cm^−1^, and 885 cm^−1^ [30, 31]) were also identified.

The peaks, which are detected at 2945 and 2868 cm^−1^ (CH2 stretching), 1096, 1096, and 962 cm^−1^ (O-C vibrations), and 1243 and 1177 cm^−1^ (C-O-C stretching), can approve the successful development of PCL/CS scaffold. Moreover, the band at 1732 cm^−1^ was related to the C=O stretching of PCL. The spectrum of MTA blended scaffold (PCL/CS/MTA) offered a slight shifting of position in the characteristic bands of OH groups (from 3430 to 3436 cm^−1^ and from 1177 to 1185 cm^−1^) due to the hydrogen bond formation between the MTA and the OH and NH_2_ groups of PCL and CS, respectively. Moreover, XRD analysis confirmed that the synthesis process applied to achieve the PCL/CS/MTA was successful. As shown in Figure 1(b), the crystalline nature of the prepared scaffold was characterized, and the pattern of PCL/CS showed diffraction peaks at 2*θ* = 21, which can be attributed to the (110, 200) plans of the PCL component, respectively. The appearance of new peaks in the PCL/CS/MTA pattern confirmed the presence of MTA in the developed scaffold. Moreover, the XRD pattern of MTA showed firm peaks at 23.580 and 26.587° 2*θ*, representing tantalum oxide. Other than tantalum oxide, the major components in the MTA were calcium silicate oxide and calcium silicate, which have characteristic peaks at 34.2 and 29.6° 2*θ*.

**Figure 2:**
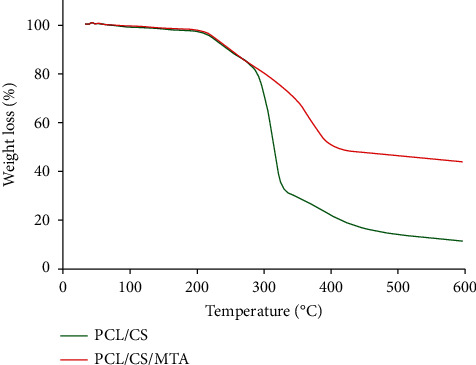
Thermogravimetric analysis (TGA) curves of PCL/CS and PCL/CS/MTA.

**Figure 3:**
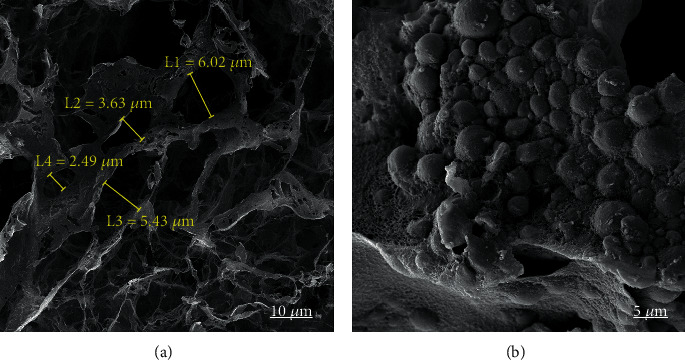
SEM images of (a) PCL/CS/MTA scaffold and (b) adhesion of hDPSCs on PCL/CS/MTA scaffold after 7 days of culture.

To confirm the successful loading of MTA in the scaffold, thermal degradation of PCL/CS and PCL/CS/MTA were carried out by thermogravimetric analysis (TGA) (Figure 2). This figure shows that the evaporation of water and residual solvent caused the weight loss below 120°C. Moreover, the second stage, between 200°C and 300°C, was due to the polymer's thermal degradation. The third stage was detected from 300°C to 400°C and ascribed to the polymeric material carbonization. Additionally, a lower weight loss was shown compared to the scaffold without MTA due to the higher melting point of MTA. The content of MTA can be calculated based on the TGA curve. Here, this value was 30% for PCL/CS/MTA. Also, the surface morphology of PCL/CS/MTA scaffolds was imaged via SEM (Figure 3(a)).  Figure 3(b) shows that hDPSCs were attached in PCL/CS/MTA scaffolds.

**Figure 4:**
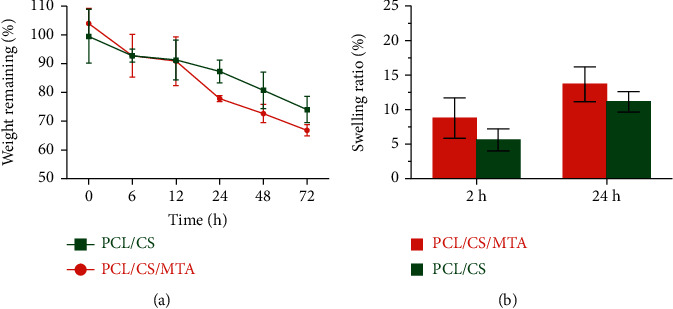
(a) *In vitro* degradation profile of the PCL/CS and PCL/CS/MTA scaffolds at pH 7.4. (b) Swelling degree (%) of PCL/CS and PCL/CS/MTA scaffolds at different time intervals.

The degradation profiles of PCL/CS and PCL/CS/MTA scaffolds were assessed, and the results are demonstrated in Figure 4(a). Based on these results, fabricated scaffolds were degraded by time passing. After the addition of MTA, the degree of degradation increased over time related to the high porosity created by the MTA, which leads to the diffusion of water molecules into the hydrogel structure, and rapid weight loss occurs. As shown in Figure 4(a), the weight remaining around 26% and 34% was observed for the PCL/CS/MTA and PCL/CS scaffolds at pH 7.4, respectively.

Moreover, the swelling index of developed scaffolds is shown in Figure 4(b). The results showed that incorporating MTA in the scaffold increased the swelling ratio due to the high porosity in the structure.

### 3.2. PCL/CS/MTA Scaffolds Increased Proliferation of hDPSCs

**Figure 5:**
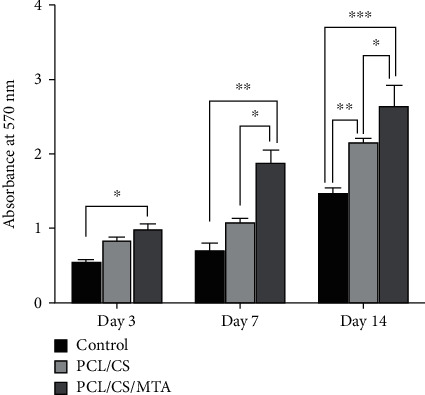
MTT assays for evaluation of hDPSC viability on PCL/CS and PCL/CS/MTA scaffolds cultured for 3, 7, and 14 days. ^∗^*P* ≤ 0.05, ^∗∗^*P* ≤ 0.01, and ^∗∗∗^*P* ≤ 0.001.

The effect of PCL/CS and PCL/CS/MTA on the proliferation and cellular viability of hDPSCs was evaluated by MTT assay after 3, 7, and 14 days. The results demonstrated that cell proliferation increased by time lapsing (Figure 5). Cell proliferation was significantly higher in PCL/CS/MTA scaffolds in all experiment times. The significant difference between PCL/CS and PCL/CS/MTA was observed on days 7 and 14. According to findings, it could be said that the PCL/CS/MTA scaffolds have positive effects on hDPSC proliferation.

### 3.3. PCL/CS/MTA Scaffolds Increased the Expression of Angiogenic Genes and Proteins in hDPSCs

**Figure 6:**
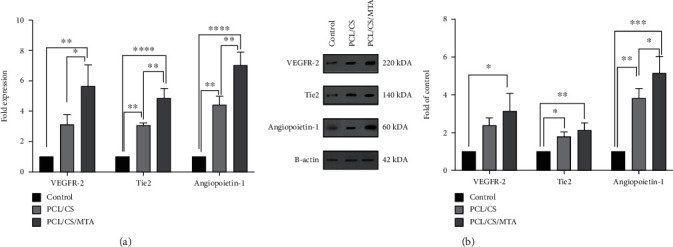
(a) Expression levels of VEGFR-2, Tie2, and Angiopoietin-1 genes in hDPSCs seeded on PCL/CS and PCL/CS/MTA scaffolds after 7 days. (b) Immunoblotting assay for the proteins of these markers showed increased levels of these proteins in hDPSCs, too. ^∗^*P* ≤ 0.05, ^∗∗^*P* ≤ 0.01, ^∗∗∗^*P* ≤ 0.001, and ^∗∗∗∗^*P* ≤ 0.000.

The modifications of angiogenic factors in hDPSCs with/without scaffolds were evaluated both in gene and protein levels (Figure 6). According to qRT-PCR results, the expression levels of VEGFR-2, Tie2, and Angiopoietin-1 were drastically increased in cultured hDPSCs on PCL/CS/MTA scaffolds comparing cells without scaffolds (*P* < ∗∗, <∗∗∗∗, and <∗∗∗∗, respectively) (Figure 6(a)). For validation of gene expression experiments, angiogenic factors were analyzed at the proteomic level. Based on western blotting findings, the seeding of hDPSCs on MTA-based scaffolds significantly increased the expression of VEGFR-2, Tie2, and Angiopoietin-1 compared to the control group (*P* < ∗, <∗∗, and <∗∗, respectively) (Figure 6(b)).

## 4. Discussion

Regeneration concepts of dentin-pulp complex by promoting bleeding in root canal space were first described by Obstby in 1960. Briefly, in this theory, reconstruction of vascular network or revascularization in root canal induced tissue regeneration [8]. In REPS, revascularization of root canal space is essential to repair and reconstruct the dentin-pulp complex [32]. Without an adequate blood supply, the regeneration procedure cannot be accomplished, and necrotic or fibrotic tissue can be formed [33]. The present study is aimed at constructing PCL/CS/MTA scaffolds and evaluating their ability to induce angiogenesis in hDPSCs. According to our results, fabricated PCL/CS/MTA scaffolds increased the expression of angiogenic markers in hDPSCs both in genomic and proteomic levels. In addition, these scaffolds provide an appropriate environment for the adhesion and proliferation of these cells.

Recently, it has been well documented that the scaffold materials and their physicochemical interaction play an essential role in tissue engineering because they provide structural support for stem cells and mimic the extracellular matrix [34, 35]. In this regard, it has been validated that the development of different scaffolds with PCL and other suitable materials with desirable regenerative properties that mimic the extracellular matrix can provide a 3D environment for cell adhesion, proliferation, and differentiation [8, 36]. Moreover, the mineralization potential of PCL-based scaffolds is considerable in dentin regeneration as it stimulates odontogenic differentiation of hDPSCs [12]. CS polymer is applied in dentin-pulp complex regeneration due to the mineralization induction, low solubility, chelating agent, anti-inflammation, and antimicrobial effects [17, 18, 37]. Therefore, it may be the case that the combination of these synthesized and natural polymers can provide an appropriate structure for the regeneration of the dentin-pulp complex.

In the current study, the addition of MTA to PCL/CS-based scaffolds significantly increased the proliferation of hDPSCs on all experiment days. For authorization of this finding, it can be mentioned that Thalita et al. loaded MTA in collagen-based scaffolds for bone regeneration induction in osteoblasts. They showed increased levels of cell viability and mineralization induction [38].

The angiogenic induction of different calcium silicate-based cements showed that PMTA (ProRoot MTA), RMTA (RetroMTA), and MMTA (MicroMega MTA) increased the mRNA expression of VEGF, FGF-2, and Ang-1. However, these increases were higher in the PMTA group [21]. Therefore, PMTA was loaded in PCL/CS background in the current study.

It is shown that the expression of VEGF receptors activates molecular and cellular cascades to vascularization, and it is necessary to induce angiogenesis and differentiation in hDPSCs [39, 40]. In this regard, Youssef et al. evaluated angiogenic induction in hDPSCs treated with MTA. Their results showed upregulated levels of VEGF marker in the MTA-treated group compared with the control group [41]. Moreover, Chou et al. demonstrated that the secretion of Angiopoietin-1, as another essential factor for angiogenesis, elevated through the contact of hDPSCs with calcium silicate-based cements like PMTA [42]. It has been well reported that hDPSCs can secrete proangiogenic factors such as VEGF, FGF-2, PDGF, MMP-9, and IGF-1 [43]. In the current study, the expression levels of VEGFR-2, Tie2, and Angiopoietin-1 in hDPSCs were evaluated to confirm the angiogenic induction potential of fabricated PCL/CS/MTA scaffolds. The obtained results demonstrated the successful fabrication of these scaffolds. Moreover, the enhanced angiogenic capacity of the PCL/CS backbone after the addition of MTA showed this structure's efficacy. However, this study was the initial step, and further studies for clinical application of these MTA-based scaffolds should be conducted.

## 5. Conclusion

MTA has potential regenerative effects as a root repair material and can be a promising component for future regenerative dentistry. Also, this material does not provide structure as same as the extracellular matrix, which is essential for tissue regeneration. Therefore, the construction of MTA-based scaffolds can be a promising alternative for regenerative endodontic procedures. In summary, in the current study, the MTA-based scaffolds were fabricated successfully and provided an appropriate structure for the adhesion and proliferation of hDPSCs. Moreover, the angiogenic potential of hDPSCs seeded on the PCL/CS/MTA scaffold was increased. On one more general note, it can be said that the addition of MTA to PCL/CS backbone can increase the angiogenic induction, and these results can be applied in the future for regenerative endodontic procedures.

## Figures and Tables

**Table 1:** List of primers.

Genes	Sense	Antisense	Tm
VEGFR-2	CCAGCAAAAGCAGGGAGTCTGT	TGTCTGTGTCATCGGAGTGATATCC	60
Tie2	ATAGGGTCAAGCAACCCAGC	GCTGGTTCTTCCCTCACGTT	60
Angiopoietin-1	GGACAGCAGGAAAACAGAGC	CACAAGCATCAAACCACCAT	63
*β*-Actin	AGTGTGACGTTGACATCCGT	TGCTAGGAGCCAGAGCAGTA	60

## Data Availability

All data generated and/or analyzed during this study are included in this published article. The datasets used and/or analyzed during the current study are available from the corresponding author on reasonable request.
